# Galactomannan Catabolism Conferred by a Polysaccharide Utilization Locus of *Bacteroides ovatus*: ENZYME SYNERGY AND CRYSTAL STRUCTURE OF A β-MANNANASE*[Fn FN2]

**DOI:** 10.1074/jbc.M116.746438

**Published:** 2016-11-21

**Authors:** Viktoria Bågenholm, Sumitha K. Reddy, Hanene Bouraoui, Johan Morrill, Evelina Kulcinskaja, Constance M. Bahr, Oskar Aurelius, Theresa Rogers, Yao Xiao, Derek T. Logan, Eric C. Martens, Nicole M. Koropatkin, Henrik Stålbrand

**Affiliations:** From the ‡ Department of Biochemistry and Structural Biology, Lund University P. O. Box 124, S-221 00 Lund, Sweden and; the § Department of Microbiology and Immunology, University of Michigan Medical School, Ann Arbor, Michigan 48109

**Keywords:** carbohydrate metabolism, enzyme kinetics, glycobiology, glycoside hydrolase, Gram-negative bacteria, structure-function, Bacteroides ovatus, galactomannan, human gut bacteria, polysaccharide utilization loci

## Abstract

A recently identified polysaccharide utilization locus (PUL) from *Bacteroides ovatus* ATCC 8483 is transcriptionally up-regulated during growth on galacto- and glucomannans. It encodes two glycoside hydrolase family 26 (GH26) β-mannanases, *Bo*Man26A and *Bo*Man26B, and a GH36 α-galactosidase, *Bo*Gal36A. The PUL also includes two glycan-binding proteins, confirmed by β-mannan affinity electrophoresis. When this PUL was deleted, *B. ovatus* was no longer able to grow on locust bean galactomannan. *Bo*Man26A primarily formed mannobiose from mannan polysaccharides. *Bo*Man26B had higher activity on galactomannan with a high degree of galactosyl substitution and was shown to be endo-acting generating a more diverse mixture of oligosaccharides, including mannobiose. Of the two β-mannanases, only *Bo*Man26B hydrolyzed galactoglucomannan. A crystal structure of *Bo*Man26A revealed a similar structure to the exo-mannobiohydrolase *Cj*Man26C from *Cellvibrio japonicus,* with a conserved glycone region (−1 and −2 subsites), including a conserved loop closing the active site beyond subsite −2. Analysis of cellular location by immunolabeling and fluorescence microscopy suggests that *Bo*Man26B is surface-exposed and associated with the outer membrane, although *Bo*Man26A and *Bo*Gal36A are likely periplasmic. In light of the cellular location and the biochemical properties of the two characterized β-mannanases, we propose a scheme of sequential action by the glycoside hydrolases encoded by the β-mannan PUL and involved in the β-mannan utilization pathway in *B. ovatus.* The outer membrane-associated *Bo*Man26B initially acts on the polysaccharide galactomannan, producing comparably large oligosaccharide fragments. Galactomanno-oligosaccharides are further processed in the periplasm, degalactosylated by *Bo*Gal36A, and subsequently hydrolyzed into mainly mannobiose by the β-mannanase *Bo*Man26A.

## Introduction

Bacterial members of the human gut microbiota encode an impressive array of glycoside hydrolases (GHs),^4^ several times more than encoded within the human genome (1). The composite metabolic activity of the microbiota acts as an extension of the human digestive system, processing much of our complex carbohydrate nutrition into host-absorbable short chain fatty acids that influence our physiology (2). Despite the number of different species present in the mammalian gut, only a few bacterial phyla dominate this environment, with members of Firmicutes and Bacteroidetes typically being the most numerous (3). Bacteroidetes is a phylum of Gram-negative bacteria that collectively encode the greatest numbers of distinct GHs in their genomes (1) and typically display substantial flexibility in their glycan utilization profiles, a feature that allows them to persist despite normal fluctuations in an individual's dietary habits (4–6). Many Bacteroidetes have been shown to have varying sets of polysaccharide utilization loci (PULs), each coding for a set of proteins involved in the recognition/binding, hydrolysis, and internalization of a specific type of carbohydrate substrate (7). Understanding the variations between different species and how they utilize various carbohydrates will increase our knowledge of how dietary glycans affect the gut microbiota and contribute to the development of new prebiotics.

*Bacteroides ovatus* is a common human gut bacterium capable of degrading and growing on several complex plant cell wall polysaccharides, such as hemicellulosic xylan- (8–10) and β-mannan-based dietary fibers (11). A study by Martens *et al.* (10) highlighted the metabolic diversity among *Bacteroides* species showing that, in contrast to the mucin-degrading *Bacteroides thetaiotaomicron,* the *B. ovatus* type strain ATCC 8483 harbors several PULs for utilization of hemicellulosic polysaccharides. Among the PULs there was a putative β-mannan PUL, which is transcriptionally up-regulated when *B. ovatus* is grown on locust bean gum (LBG) galactomannan or konjac glucomannan (KGM). *B. ovatus* has been shown to have cell-associated β-mannanase activity (12), although the identity of the corresponding enzyme(s) is not known. Some of the proteins encoded within the *Bacteroides* PULs dedicated to α-mannan (13), xylan (9, 14, 15), and xyloglucan utilization (16) have recently been characterized, demonstrating the diversity in the proteins and enzymes encoded within distinct PULs. The functional mechanisms of β-mannan PULs are, however, much less understood. This is therefore the focus of this work, using *B. ovatus* (10) as the object of study.

β-Mannans are a group of hemicellulosic polysaccharides consisting of a β-1,4-linked polymannose backbone, which may contain other sugars and chemical groups. Ivory nut mannan (INM) is a linear homomannan, whereas KGM has a partially acetylated backbone containing glucose as well as mannose units (17, 18). Seed galactomannans, such as LBG and guar gum, have α-1,6-galactosyl substitutions and are commonly used as viscosity-enhancing food additives (19, 20). To break down these β-mannans, the main GHs required are α-galactosidases, which remove the galactose substitutions, and β-mannanases, which cleave the backbone (21). β-Mannanases have so far been found in the clan GH-A families GH5, GH26, and GH113, as notated in the carbohydrate active enzymes (CAZy) database (22). Clan GH-A enzymes share a (β/α)_8_-barrel fold, a conserved retaining catalytic mechanism, and essential catalytic residues (nucleophile and acid/base (21)). β-Mannanases generally have an active site cleft with a varied number of sugar-binding subsites (−2, −1, +1, +2, etc.), with the reducing sugar being located in the + subsites and the target mannosidic bond connecting the sugars in subsites −1 and +1. Conservation is greatest within the −1 subsite where the catalytic residues are positioned. Although several β-mannanases have been characterized, including 3D structures of members of GH26 (23–29) as well as the other two families, only recently have a few studies been published on the characterization of β-mannanases from human gut bacteria (30–32). So far, no crystal structure of a β-mannanase from the human gut microbiota has been determined.

By homology to the starch-utilization protein system (Sus) of *B. thetaiotaomicron* (33), the *B. ovatus* β-mannan PUL was predicted to encode cell-associated Sus-C/D-like transport and saccharide recognition proteins and a hybrid two-component system (HTCS) sensor protein that was shown to bind unsubstituted manno-oligosaccharides (10). The PUL was recently shown to have homologues in other *B. ovatus* and *Bacteroides xylanisolvens* strains (34). Furthermore, the PUL was predicted to encode putative GHs required for β-mannan degradation, including a family GH36 α-galactosidase (*Bo*Gal36A) recently characterized by us (34).

The aim of this study is to reveal the significance of this *B. ovatus* ATCC 8483 β-mannan PUL for galactomannan utilization and to investigate the structure-function relation and role of the putative GHs of the PUL, *i.e.* two GH26 β-mannanases, *Bo*Man26A and *Bo*Man26B. In addition, a second putative β-mannan PUL was discovered in the *B. ovatus* genome. Growth studies with strains where genes from either or both of these PULs have been deleted highlights the importance of the first PUL (*bacova_02087–02097*) for galactomannan utilization. The two β-mannanase genes are cloned, and the recombinant proteins are characterized. The crystal structure of the β-mannanase *Bo*Man26A reveals the molecular details governing enzymatic activity. Finally, determination of the cellular location for the β-mannanases *Bo*Man26A and *Bo*Man26B and the α-galactosidase *Bo*Gal36A allowed us to propose a model for catabolic galactomannan degradation by *B. ovatus* conferred by PUL *bacova_02087–02097.*

## Results

### 

#### 

##### Two Putative β-Mannan PULs in B. ovatus

**FIGURE 1.**
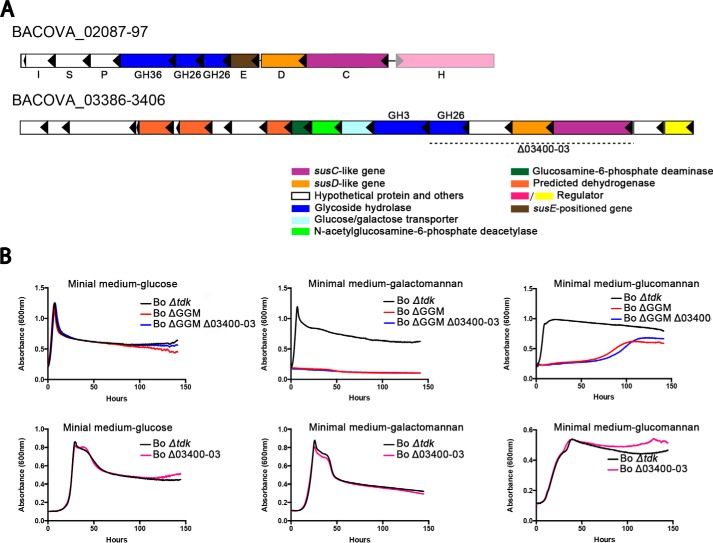
*A,* gene organization of the two investigated β-mannan-related PULs of *B. ovatus* ATCC 8483. The *color code* marks putative protein functions. The glycoside hydrolase genes have been labeled according to which family they belong, with the two GH26 β-mannanases (*Bo*Man26A and *Bo*Man26B) from *bacova_02087–97* (coded by *bacova_02092–93*) being the focus of this study. The gene for α-galactosidase *Bo*Gal36A is marked *GH36*. The genes for the SusD-like and the SusE-positioned proteins are marked *D* and *E*, respectively. The putative functions for the other genes in *bacova_02087–97* have been labeled as follows: isomerase (*I*), symporter (*S*), mannoglucosyl phosphorylase (*P*), SusC (*C*), and HTCS regulator (*H*). The *bacova_03386–03406* was identified as a potential β-mannan PUL due to containing a gene encoding a putative GH26 β-mannanase (*bacova_03400*). In the mutant strain, *B. ovatus* ΔGGM *bacova_02087–02096* is deleted. The deletion (*bacova_03400–03403*) in strain *B. ovatus* Δ03400-03 is shown with a *dotted line. B,* cultivation of *B. ovatus* deletion mutants were described in *A*. Δ*tdk* denotes the parental strain used to create the deletions in the PULs. The *B. ovatus* strain ΔGGM Δ03400-03 with both the above-described deletions was also constructed. Growth curves represent the average of six parallel replicate cultures using glucose, LBG (galactomannan), or KGM (glucomannan) as sole carbon source.

As described above, a putative β-mannan PUL was previously identified in *B. ovatus* ATCC 8483 (*bacova_02087–02097*) and shown to be transcriptionally up-regulated when galactomannan or glucomannan was included in the culture medium (10, 34). This PUL contains the genes *bacova_02092* and *bacova_02093*, which encode the putative GH26 β-mannanases *Bo*Man26A and *Bo*Man26B, respectively. To investigate the function of the *bacova_02087–02097* PUL, it was deleted, thus creating the strain *B. ovatus* ΔGGM (Fig. 1). We furthermore performed an *in silico* analysis of the *B. ovatus* ATCC 8483 genome. By BlastP searches using characterized bacterial GH5, GH26, and GH113 β-mannanases, we identified a third putative GH26 β-mannanase gene (*bacova_03400*), which we discovered to be part of a second potential β-mannan related PUL (*bacova_03386–03406*) (Fig. 1). This PUL in addition has genes coding for a putative GH3 β-glucosidase (*bacova_03399*), as well as SusC and SusD homologues (*bacova_03402* and *03403*) and other putative proteins. No other gene encoding a putative β-mannanase was found in the genome of *B. ovatus* ATCC 8483. To investigate the potential function of this new PUL in relation to β-mannan catabolism, the DNA fragment *bacova_03400–03403* (including the genes for the GH26 and the susC/D-like proteins) was deleted creating the strain *B. ovatus* Δ3400-03 (Fig. 1). A double deletion strain containing both the aforementioned deletions was also created (strain *B. ovatus* ΔGGMΔ03400-03, Fig. 1).

To investigate the potential function of the two PULs containing putative GH26 β-mannanases, the deletion strains and the isogenic parent (*B. ovatus* Δ*tdk,* see under “Experimental Procedures”) were grown on LBG galactomannan and KGM. *B. ovatus* Δ*tdk* grew well on LBG, although the ΔGGM strain did not (Fig. 1). Deletion of *bacova_03400–03403*, alone or in combination with *bacova_02087–02096*, did not affect growth compared with strains Δ*tdk* or ΔGGM, respectively (Fig. 1). Similarly *B. ovatus* Δ*tdk* also grew well on KGM, and deletion of *bacova_03400–03403* had no effect, but growth of *B. ovatus* ΔGGM was dramatically hampered (Fig. 1). These results clearly suggest that *bacova_02087–02097* is the main PUL conferring growth on galactomannan and also significantly contributes to growth on glucomannan. However, the *bacova_03386–03406* PUL is not significantly contributing to gluco- or galactomannan growth, and its role remains unclear. To make a functional assessment of the predicted surface glycan-binding proteins of the GGM PUL, Bacova_02095 and Bacova_02094 with predicted functional similarity to SusD and SusE proteins were recombinantly produced, and their affinity for galactomannan and glucomannan was shown by affinity electrophoresis (supplemental Fig. S1). Both proteins were retarded in the presence of the mannan polysaccharides, although BT1043, a SusD that targets mucosal glycans (35, 36), was not.

##### Bioinformatic Analysis and Cellular Location

Bioinformatic analysis of the *Bo*Man26A and *Bo*Man26B protein sequences was conducted. The LipoP server predicted the presence of a signal peptidase II cleavage site for both *Bo*Man26A and *Bo*Man26B, indicating that both of these enzymes would be membrane-anchored through the sulfhydryl group of Cys, after cleavage by signal peptidase II (supplemental Fig. S2). *Bo*Man26A was in addition predicted to have a signal peptidase I cleavage site. Based on these predictions, the genes *bacova_02092* and *bacova_02093* were cloned, and the proteins were expressed without their N-terminal signal sequences and purified (supplemental Fig. S3).

**FIGURE 2.**
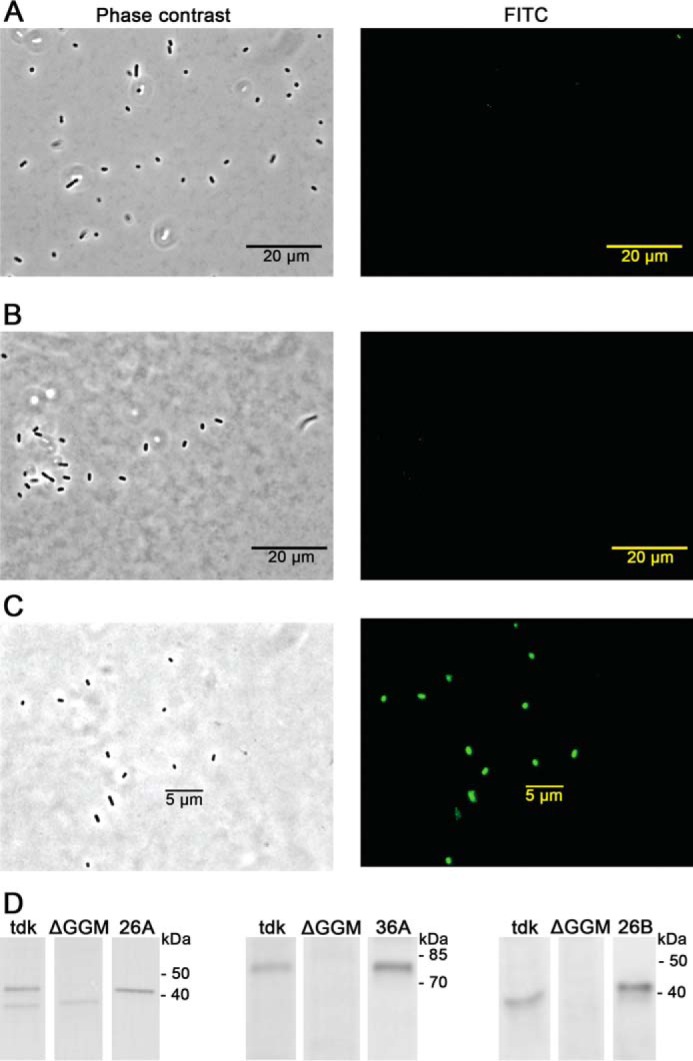
Cellular location analyses of *Bo*Man26A (*A*), *Bo*Gal36A (*B*), and *Bo*Man26B (*C*) are shown. Cells fixed with formaldehyde were stained with antibodies generated against the three *B. ovatus* enzymes and labeled with fluorescently labeled goat anti-rabbit antibodies. The cells were imaged using phase contrast microscopy (63) and fluorescence imaging. *D,* Western blottings of *Bo*Man26A, *Bo*Gal36A, and *Bo*Man26B with *B. ovatus* Δ*tdk* and ΔGGM cell extracts and pure protein (marked *26A*, *36A*, and *26B*, respectively). Each enzyme was run on a single blot, with smaller spaces indicating where the image had been spliced to remove inappropriate concentrations. Sizes of the relevant bands from the ladder have been labeled in kDa. The *left set* (*3 lanes*) is stained with *Bo*Man26A-specific primary antibodies, and the following samples were applied in the SDS-polyacrylamide gels (from *left to right*): Δ*tdk* and ΔGGM 1:20 dilutions from original cell extracts and *Bo*Man26A 10 ng. The *middle set* is stained with *Bo*Gal36A-specific primary antibodies and contains the following applied samples: Δ*tdk* and ΔGGM 1:5 dilutions from original cell extracts and *Bo*Gal36A 100 ng. The *right set* is stained with *Bo*Man26B-specific primary antibodies and contains the following applied samples: Δ*tdk* and ΔGGM 1:1 dilutions from original cell extracts and *Bo*Man26B 5 ng.

To further investigate the mechanism of β-mannan degradation by *B. ovatus*, we determined the cellular location of *Bo*Man26A, *Bo*Man26B, and the α-galactosidase *Bo*Gal36A, the latter of which carries a signal peptidase I site but no membrane-attachment motif (34). Custom antibodies to the three recombinant proteins were used to detect surface expression in formaldehyde-fixed non-permeabilized *B. ovatus* grown in minimal media containing LBG galactomannan as a sole carbon source. Although Western blotting demonstrated that all three antibodies recognize the proteins in cell extracts, only the anti-*Bo*Man26B-labeled whole cells displayed fluorescence, suggesting that *Bo*Man26B is expressed on the cell surface and both *Bo*Man26A and *Bo*Gal36A are located in the periplasm (Fig. 2). The *B. ovatus* mutant strain (ΔGGM) lacking most of the β-mannan PUL was used as a negative control and did not display specific immunofluorescence with any of the antibodies, although some cross-reactivity with an unknown protein was visible in the Western blotting for the *Bo*Man26A antibodies (Fig. 2).

##### Stability and pH Optima

*Bo*Man26A and *Bo*Man26B were optimally active at pH 6.5–7.5 and 6–6.5 at 37 °C, respectively. *Bo*Man26A was stable at 4 °C for 6 months and at 37 °C for 24 h, with only a minor decrease of activity at 45 °C. *Bo*Man26B was stable for 2 months at 4 °C and 24 h at 30 °C, but it only retained 50% activity after 24 h at 37 °C and quickly lost activity at 45 °C (supplemental Fig. S4). Because both *Bo*Man26A and -B were optimally active around pH 6.5 (supplemental Fig. S4*B*), this was the standard pH value of all substrate incubations used for characterization.

##### Catalytic Properties and Product Profiles

High performance anion exchange chromatography with pulsed amperometric detection (HPAEC-PAD) analysis showed that *Bo*Man26A was able to hydrolyze all tested mannan polysaccharides and the oligosaccharides mannotriose to mannohexaose (M3–M6), but not mannobiose (M2), Avicel cellulose, or xylan. For LBG, the specific activity was 301 ± 4.5 katal/mol, and the activity for KGM was similar, with a 100-fold decrease in activity for INM (Table 1). This specific activity on LBG is similar to other GH26 mannanases (27, 30). The specific activity on guar gum was very low, ∼360-fold lower than for LBG (Table 1), and based on HPAEC-PAD comparisons, the activity on partially hydrolyzed guar gum and galactoglucomannan (GGM) was barely detectable (data not shown). The oligosaccharide kinetics for *Bo*Man26A showed catalytic efficiencies (*k*_cat_/*K_m_*) for mannotetraose (M4) to M6 hydrolysis around 10^7^ m^−1^ min^−1^, while being more than 2000-fold slower for M3 (Table 2). The M3–M6 hydrolysis profiles for *Bo*Man26A and *Bo*Man26B were similar with the dominant end product being M2 for both enzymes. M6 hydrolysis generated primarily M4 and M2, with a minor amount of M3. Mannopentaose (M5) generated M3 and M2 as initial products; M4 was only hydrolyzed into M2, and M3 was hydrolyzed into M2 and mannose.

**TABLE 1:** **Relative specific activities (%) of *Bo*Man26A and *Bo*Man26B as a percentage of LBG-specific activity**

Substrates	Relative specific activity, as compared with LBG (%)*^a^*	
	*Bo*Man26A	*Bo*Man26B
LBG	100 ± 4.1	100 ± 2.1
KGM	174 ± 41	65.4 ± 4.1
INM	1.93 ± 0.23	1.85 ± 0.28
Guar	0.28 ± 0.13	86.8 ± 6.2

*^a^* Relative specific activity (%) for each enzyme to various mannans with LBG for each enzyme set to 100%. LBG-specific activity is 301 ± 4.5 katal/mol for *Bo*Man26A and 30 ± 0.9 katal/mol for *Bo*Man26B.

**TABLE 2:** **Oligosaccharide kinetics for *Bo*Man26A and *Bo*Man26B**

Substrate	*k*_cat_/*K_m_*	
	*Bo*Man26A	*Bo*Man26B
	*m*^−*1*^ *min*^−*1*^	
M6	1.18 × 10^7^ ± 3.88 × 10^6^	2425 ± 254
M5	1.48 × 10^7^ ± 2.21 × 10^6^	ND*^a^*
M4	8.44 × 10^6^ ± 1.62 × 10^6^	ND
M3	3.6 × 10^3^ ± 542	ND

*^a^* ND means not determined.

The specific activity for *Bo*Man26B on LBG was 30 ± 0.9 katal/mol. It had a similar activity on KGM and guar gum but about 60-fold lower activity on INM (Table 1). No detectable activity could be seen when products were analyzed with HPAEC-PAD for Avicel cellulose or xylan, but it hydrolyzed all tested manno oligo- and polysaccharides (M3–M6 and INM, LBG, guar gum, KGM, and GGM, respectively), with the exception of M2. Oligosaccharide kinetics for *Bo*Man26B yielded a *k*_cat_/*K_m_* of 2425 ± 254 m^−1^ min^−1^ for M6 (Table 2). When *Bo*Man26B was incubated with M3–M5 at the same enzyme and substrate concentration as for M6, the conversion was too low to obtain reliable kinetic data, suggesting even lower catalytic efficiency and that *Bo*Man26B has at least six contributing subsites.

**FIGURE 3.**
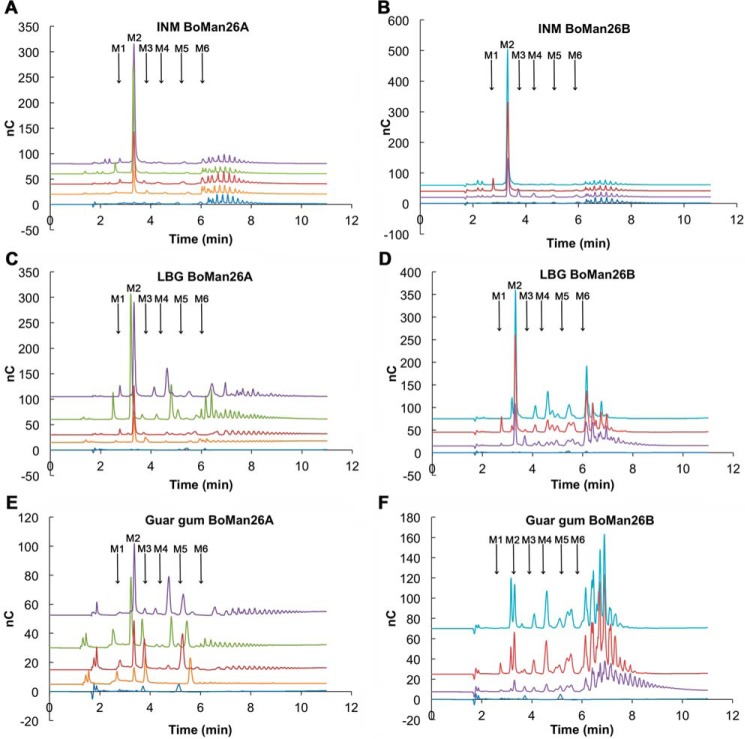
**Hydrolysis products for *Bo*Man26A after 24 h (*purple*), 3 h (*green*), 20 min (*red*), and 2 min (*orange*) and *Bo*Man26B after 24 h (*light blue*), 3 h (*red*), and 15 min (*purple*) for INM (*A* and *B*), LBG (*C* and *D*), and guar gum (*E* and *F*).** The blank (*dark blue*) runs along the *x* axis. The position of the *M1–M6* peaks have been marked according to the standards. The other visible peaks are unidentified oligosaccharides.

The dominant product from INM, both initially and over the course of hydrolysis, was M2 for both *Bo*Man26A and *Bo*Man26B (Fig. 3, *A* and *B*). *Bo*Man26B also produced manno-oligosaccharides with a degree of polymerization (DP) of 3–5 as minor initial products. Both *Bo*Man26A and *Bo*Man26B fragmented LBG and guar galactomannans into oligosaccharides of approximately DP 2 and higher and significantly beyond DP 6 (Fig. 3). These product profiles suggest capability of endo-action on galactomannans for both *Bo*Man26A and *Bo*Man26B, yet the profiles differed between the two. From LBG, the initial release of M2 as a dominant product was significant for *Bo*Man26A, with simultaneous production of some fragments with an estimated DP of 6 and higher. At longer incubation times, smaller fragments appeared with a significant product at approximately DP 4 (Fig. 3*C*). *Bo*Man26B instead initially released a range of significant oligomeric products and during the course of hydrolysis, although M2 was a major product for later time points (Fig. 3*D*). For guar gum hydrolysis, the differences in product profiles were more pronounced. *Bo*Man26A released a few significant smaller oligosaccharides (DP 2–6, including M2), and some fragments were estimated to DP 6 and higher. In contrast, *Bo*Man26B significantly released fragments higher than DP 6 with only minor production of oligosaccharides of approximately DP 2–6 (including M2) (Fig. 3, *E* and *F*).

##### Productive M5-binding Modes of BoMan26A

**FIGURE 4.**
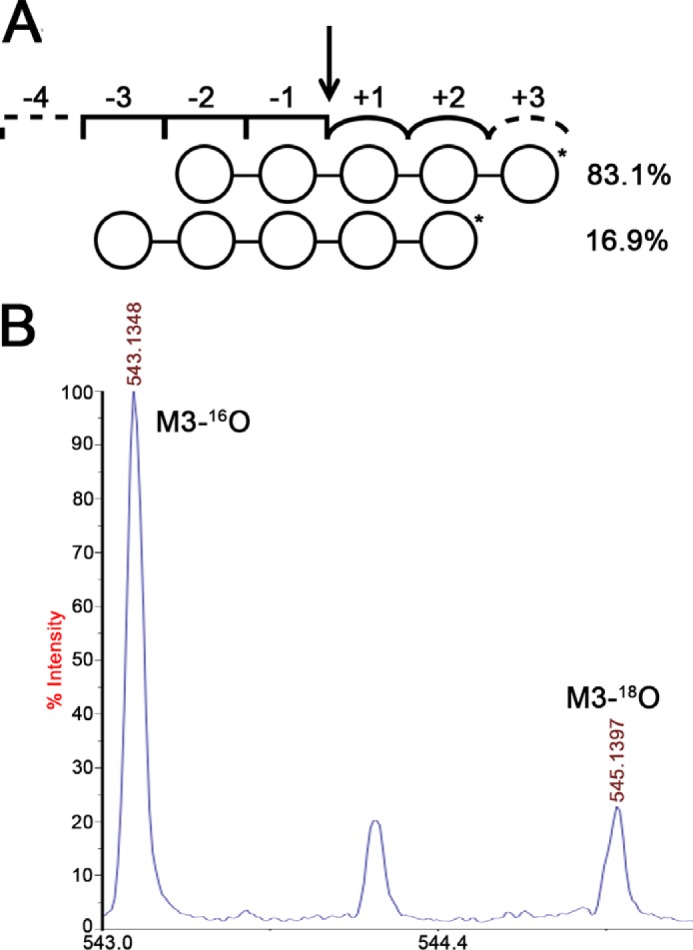
*A,* scheme representing the two productive binding modes of M5 hydrolysis by *Bo*Man26A. Based on the data in *B,* the distribution of the productive binding modes were 83.1 and 16.9%. The M5 hydrolysis was 18%. *B,* MALDI-TOF spectrum showing the *M3-^16^O* and *M3-^18^O* peaks after the M5 hydrolysis. The *middle peak* is a natural M3 ^13^C isotope.

Oligosaccharide hydrolysis by *Bo*Man26A was studied further using ^18^O labeling. M5 hydrolysis exclusively formed M3 and M2. The preferable productive binding mode could thus be from subsite −3 to +2 or from −2 to +3. To differentiate between these two binding modes, the ratio of ^16^O/^18^O-labeled products was determined in accordance with a previously established method (37, 38). The ratio of unlabeled to labeled M3 was determined to 4.91. Thus, during M5 hydrolysis, the enzyme preferably binds from subsite −2 to +3, but it is also clearly capable of binding from subsite −3 to +2 (Fig. 4).

##### BoGal36A and BoMan26A Synergy

*Bo*Gal36A showed galactose release from digalactosyl-mannopentaose (G2M5), although there was no detectable M2 release for *Bo*Man26A. When the enzymes were coincubated, *Bo*Man26A was able to release M2, although the galactose release of *Bo*Gal36A remained unchanged (Table 3). The same pattern could be seen for LBG; when coincubated with *Bo*Gal36A, *Bo*Man26A released M2 with almost a 3-fold higher rate (Table 3). The changes in M2 release clearly suggest that *Bo*Man26A acts in synergy with *Bo*Gal36A. Furthermore, because the rate of galactose release of *Bo*Gal36A is about the same (or even lower) when coincubated with *Bo*Man26A, this suggests a sequential action, with *Bo*Gal36A initially removing the galactose substitutions before cleavage of the oligosaccharide by *Bo*Man26A.

**TABLE 3:** **Synergy experiments using G2M5 and LBG**

	M_2_ release	Galactose release
	μ*mol/min*	μ*mol/min*
**G2M5**		
*Bo*Man26A	<0.001	<0.001
*Bo*Gal36A	<0.001	0.239 ± 0.040
*Bo*Man26A + *Bo*Gal36A	0.056 ± 0.01	0.265 ± 0.032

**LBG**		
*Bo*Man26A	0.064 ± 0.011	<0.001
*Bo*Gal36A	<0.001	0.156 ± 0.003
*Bo*Man26A + *Bo*Gal36A	0.176 ± 0.020	0.075 ± 0.002

##### Crystal Structure of BoMan26A

**FIGURE 5.**
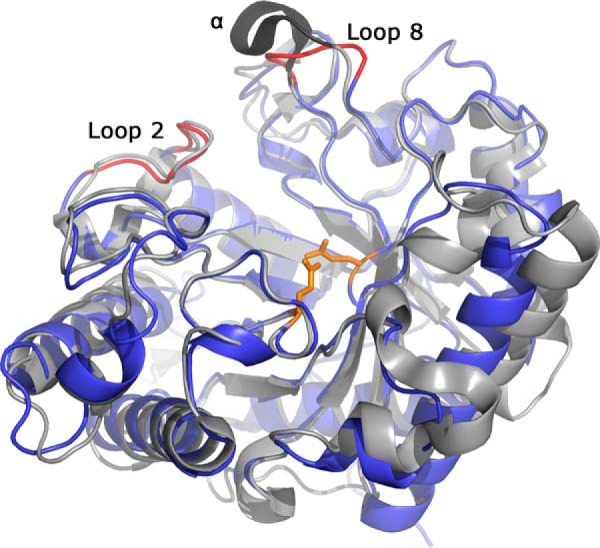
**Overview of the *Bo*Man26A 3D structure (PDB code 4ZXO, *blue*), superimposed with *Cj*Man26C (PDB code 2VX6, *gray*), looking into the active site cleft.** *Loop 2* and *loop 8* (*red*) and the α-helical turn of *Cj*Man26C (*dark gray*) have been labeled. The catalytic residues are shown and colored *orange*.

**FIGURE 6.**
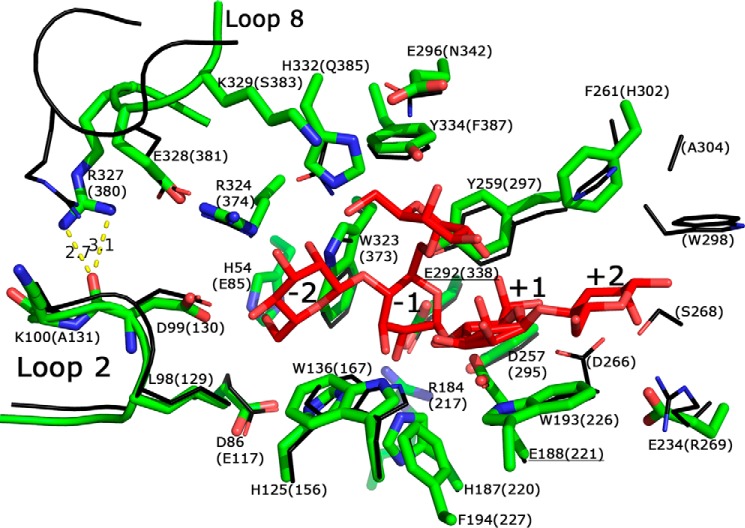
**Overview of the active site residues in *Bo*Man26A (*thick green lines*), superimposed with *Cj*Man26C (*thin black lines*) with a bound G1M4 (*red*).** The residue numbering is according to *Bo*Man26A with the corresponding residues in *Cj*Man26C in *parentheses*, including the one-letter code for the residues that differ from *Bo*Man26A. The labels for the catalytic residues are *underlined*. The residues shown are those located within 5 Å of the sugar, as well as adjacent residues from loops 2 and 8 that could be relevant for a −3 subsite. Loop 2 spans residues 86–103, and loop 8 spans residues 323–342. The subsites have been marked. Of the residues not conserved between *Bo*Man26A and *Cj*Man26C, only the following residues of *Cj*Man26C interact with the sugar: His-302, Asn-342, Gln-385, and Phe-387. The *Cj*Man26C residues beyond subsite +2 lack equivalents in *Bo*Man26A. The hydrogen bonds between Arg-237 (in *loop 8*) and the backbone oxygen of Asp-99 (in *loop 2*) are shown, and the distance is displayed in Å.

*Bo*Man26A was shown to be monomeric using native PAGE (data not shown). It has the highest sequence identity (66%) to *Bf*Man26, a GH26 β-mannanase from *Bacteroides fragilis* (31). A 3D structure of *Bo*Man26A was obtained using molecular replacement with the structure of GH26 mannanase C from *Cellvibrio japonicus* (*Cj*Man26C), the closest homologue with a solved structure (23). The obtained *Bo*Man26A structure contained one monomer (residues 30–373) in the asymmetric unit at 1.5 Å resolution (PDB code 4ZXO, see Fig. 5 and Table 4). The protein displays the expected β_8_α_8_-barrel structure, which is conserved in all GH26 enzymes, with the active site located in a cleft. Because of crystal packing, the fused His tag of one monomer is situated in the active site cleft of the adjacent monomer, potentially aiding the formation of the crystals. The His tag interacts with several of the residues that interact with the sugar residues in the complex of *Cj*Man26C with galactosyl-mannotetraose (G1M4) bound in the active site (PDB code 2VX6) (23). Five residues of the His tag were visible in the structure with weak electron densities (supplemental Fig. S5). The structure of *Cj*Man26C (23) was superposed on that of *Bo*Man26A with a root mean square deviation (r.m.s.d.) of 0.699 Å for 243 eq C_α_ atoms via PyMOL (39) (out of a total of 373 residues in *Bo*Man26A and 419 residues in *Cj*Man26C, Fig. 5). The active site cleft showed a large degree of structural conservation (Fig. 6). The catalytic residues Glu-188 (acid/base) and Glu-292 (nucleophile) are located at the ends of β-strands 4 and 7, respectively, and are conserved within GH26 (40) and other clan GH-A families.

**TABLE 4:** **Data collection and refinement statistics** Statistics for the highest resolution shell are shown in parentheses.

Resolution range (Å)	41.27–1.50 (1.55–1.50)
Space group	P2_1_2_1_2_1_
Unit cell (*a*, *b*, *c*, α, β, γ)	46.86, 79.43, 87.18, 90, 90, 90
Total reflections	234,222
Unique reflections*^a^*	52,731 (5189)
Completeness (%)	99.76 (99.27)
*R*_merge_ (*I*) (%)	6.3 (61.6)
Wilson *B*-factor	12.01
*R*_work_ (F)	0.136 (0.218)
*R*_free_ (F)	0.175 (0.259)
No. of non-hydrogen atoms	3180
Macromolecules	2798
Associated atoms*^b^*	6
Water	376
Modeled protein residues	343
Root mean square (bonds, Å)	0.010
Root mean square (angles, °)	1.21
Ramachandran favored (%)	98
Ramachandran outliers (%)	0
Clashscore*^b^*	1.99
Average *B*-factor (Å^2^)	15.1
Macromolecules	13.6
Associated atoms*^c^*	17.7
Solvent	26.1
〈*I*〉/〈σ(*I*)〉	26.6 (4.2)

*^a^* The number of non-anomalous unique reflections are shown.

*^b^* Unfavorable all-atom steric overlaps are ≤0.4 Å per 1000 atoms (58).

*^c^* This encompasses one phosphate participating in crystal contacts and a bound potassium.

Comparing the structure of *Bo*Man26A with that of *Cj*Man26C with G1M4 shows that the −2 to +1 subsites are largely conserved, with residues His-125, His-187, Trp-193, Tyr-259, and Trp-323 being conserved throughout GH26 (28). Potential interactions with a bound substrate are thus assumed to be similar to those described for *Cj*Man26C oligosaccharide interactions (23). Of the residues in *Cj*Man26C that are described as interacting with the sugar, three are not conserved in *Bo*Man26A: Gly-232, Glu-234, and His-332 (*Bo*Man26A numbering). His-332 and the corresponding residue in *Cj*Man26C (Glu-382) are at a similar distance from the −2 mannosyl, making polar interaction possible in both cases (Fig. 6). The other two differing residues, Gly-232 and Glu-234, are both located further from the sugar than their *Cj*Man26C counterparts (Asp-264 and Arg-269, respectively). Gly-232 is in an equivalent position to Asp-264, which contributes to the +2 subsite in *Cj*Man26C, but the lack of a side chain places it too far away for interaction (Fig. 6). Beyond subsite +2, the active site cleft becomes more open in *Bo*Man26A, lacking several residues equivalent to those in *Cj*Man26C (Fig. 6).

**FIGURE 7.**
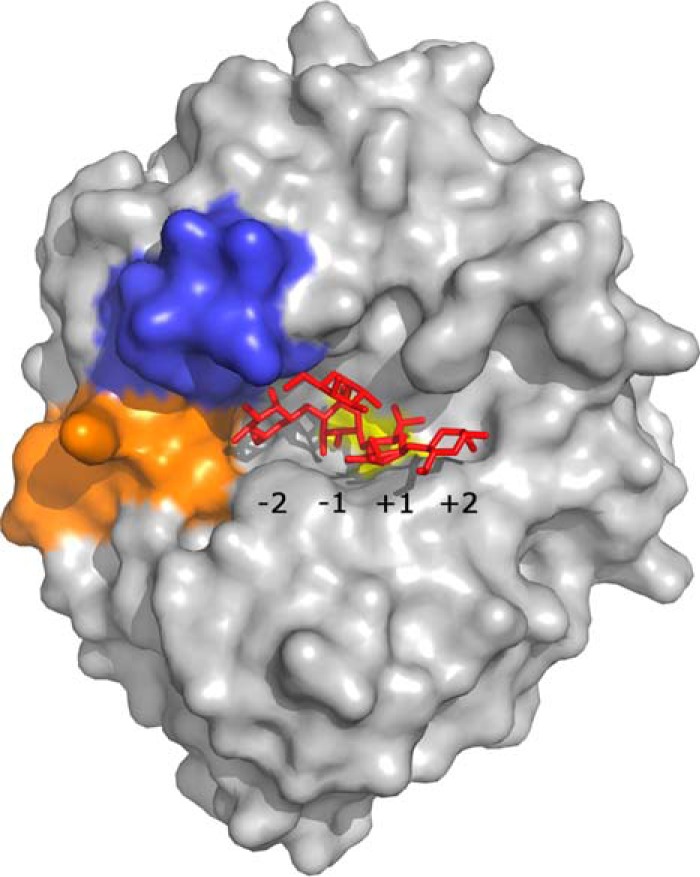
**Surface view of the active site cleft in *Bo*Man26A.** Loop 2 has been colored *orange*, loop 8 is *blue,* and the active site residues are *yellow*. The subsites have been labeled, and the oligosaccharide G1M4, superposed from the *Cj*Man26C structure, is shown as a *stick* representation.

The region beyond subsite −2 has some differences when comparing *Cj*Man26C and *Bo*Man26A. A 17-residue loop situated between β-strand 2 and α-helix 2 in *Bo*Man26A (hence referred to as “loop 2,” Fig. 7 and supplemental Fig. S2) is largely conserved with *Cj*Man26C (Fig. 6). In *Cj*Man26C, a corresponding “exo-loop” is thought to close off the possibility of a −3 subsite and confer exo-activity. This is not the case in *Bo*Man26A, where ^18^O labeling shows that occupation is possible in the −3 subsite (Fig. 4), yet the loop only contains two non-conserved residues, His-96 and Lys-100 (corresponding to Ala-127 and Ala-131 in *Cj*Man26C, respectively, see Fig. 6). Lys-100 has a relatively high *B*-factor of 30 Å^2^ (the average *B*-factor for the whole structure is 15 Å^2^), suggesting it is relatively flexible. The *Bo*Man26A loop 2 has an average *B*-factor of 10 Å^2^, which is similar to other residues surrounding the active site cleft.

There are other differences in the region beyond subsite −2 as follows: the area around a potential −3 subsite contains a short α-helix turn in *Cj*Man26C, corresponding to a loop region (hence referred to as loop 8) in *Bo*Man26A, and situated between β-strand 8 and α-helix 8 (Figs. 5 and 7 and supplemental Fig. S2). However, the approximate positions of the amino acid side chains of the α-helical turn of *Cj*Man26C and the corresponding loop 8 of *Bo*Man26A are conserved. Arg-324 and Glu-328 form a salt bridge in both structures, and although the backbone of Arg-327 is positioned differently for the two enzymes, the side chain interactions are similar, with conserved hydrogen bonding to the backbone of Asp-99 in loop 2 (Fig. 6). The average *B*-factor of loop 8 is 22.4 Å^2^ in *Bo*Man26A, which gives a ratio to the average *B*-factor of the structure of 1.49. This is a similar ratio to what is seen in the α-helical turn of *Cj*Man26C (ratio 1.46), indicating a similar level of flexibility in both loop structures. Loops 2 and 8 were compared with the corresponding loop structures of two other endo-acting GH26 β-mannanases, one from *Cellulomonas fimi* (*Cf*Man26A) (27) and a second one from *C. japonicus* (*Cj*Man26A) (26) (r.m.s.d. 1.17 and 0.95 Å for 260 and 210 C_α_ atoms when overlaid with *Bo*Man26A, respectively; data not shown). For these enzymes, the equivalent of loop 2 is shorter and thus situated further from the −2 subsite. The equivalent of loop 8 is either not visible in the structure due to flexibility (*Cj*Man26A) or is shorter (*Cf*Man26A), generating a more open region around and beyond subsite −3.

## Discussion

*Bacteroides* species residing in the human gut are known to generally encode Sus-like systems for polysaccharide utilization, which are outer membrane-associated and/or periplasmic or cytoplasmic proteins involved in polysaccharide binding, recognition, hydrolysis, and transport. However, current knowledge on GHs and systems devoted to β-mannan hydrolysis and utilization is scarce (10, 34). Only a few such studies have been devoted to distinct GHs involved in β-mannan conversion in human gut bacteria (30, 32, 41). Previous initial studies on galactomannan-degrading enzymes in *B. ovatus* indicated only cell-associated β-mannanase and α-galactosidase activity when grown on guar gum galactomannan (11, 12, 42). However, the identity of the mannanases and α-galactosidases in these early studies is not known. The genomic locus *bacova_02087-02097* was recently shown by Martens *et al.* (10) to be a PUL transcriptionally up-regulated in the presence of LBG galactomannan. Our results show that although the *B. ovatus* parental strain grows on galactomannan, the deletion strain (ΔGGM) lacking most of the *bacova_02087–02097* PUL entirely loses this capability. This strongly suggests that the *bacova_02087–02097* PUL plays a central role in the degradation of galactomannans for *B. ovatus*. Protein sequence analysis of the putative *bacova_02087–02097* PUL-encoded proteins indicated the presence of glycoside hydrolases as follows: two GH26 mannanases, *Bo*Man26A and *Bo*Man26B, in addition to the recently characterized α-galactosidase *Bo*Gal36A (34). Cellular location analysis showed that *Bo*Man26B was extracellular and membrane-attached. Also, taking the bioinformatics analysis into account, *Bo*Man26A and *Bo*Gal36A are likely located in the periplasm (Fig. 2).

### 

#### 

##### Modes of Action of BoMan26A and BoMan26B

*Bo*Man26A and *Bo*Man26B have similar product profiles using INM and the dietary fibers LBG and guar gum as substrates, differing the most using the more highly substituted guar gum (approximate degree of galactosylation: guar 1:2 and LBG 1:4, Fig. 3 (43)). Both *Bo*Man26A and *Bo*Man26B are capable of endo action, because they both fragment polymeric galactomannans into oligosaccharides of varying lengths (Fig. 3) and generate small amounts of M3 from M6 hydrolysis. In addition, ^18^O labeling showed that *Bo*Man26A was capable of binding substrate that occupied the −3 or +3 subsite, indicating an ability to bind longer substrates. Although M2 was the only product from INM hydrolysis by *Bo*Man26A, *Bo*Man26B also produced manno-oligosaccharides with a DP of 3–5 as minor products showing a detectable endo-action also on this short insoluble substrate with approximately DP 20–40 (Fig. 3) (44).

The difference in catalytic properties between *Bo*Man26A and *Bo*Man26B is more significant with galactomannans. In particular with guar galactomannan, the dramatic decrease in specific activity as compared with LBG for *Bo*Man26A suggests that it is severely hindered by galactose substituents, although *Bo*Man26B is not (Table 1). This probably contributes to the different product profiles observed for LBG and guar galactomannans. *Bo*Man26A's sensitivity to galactose substituents is also in accordance with the synergy experiments, where the hydrolysis of G2M5 and LBG increased significantly when coincubated with the α-galactosidase *Bo*Gal36A (Table 3). Several other GH26 mannanases have shown sensitivity to galactosyl side groups (21, 27, 30); *Bo*Man26B appears to be rather unusual in only being restricted to a limited extent. The specific activities and mixed product profile suggest that *Bo*Man26B is an endo-β-1,4-mannanase, which can accommodate galactosyl substituents in several subsites but releases M2 as the major product when attacking unsubstituted substrates (*i.e.* INM) or substrate regions.

The relatively low specific activity of *Bo*Man26B is comparable with at least some other β-mannanases (30, 31, 44), yet it is 10-fold lower than for *Bo*Man26A. It may be hypothesized that this comparably low specific activity for galactomannan for *Bo*Man26B potentially could be compensated for by the mannan-binding proteins (supplemental Fig. S1). This could be a similar mechanism as discussed for the Sus of *B. thetaiotaomicron* (45). In this system the extracellular starch-cleaving enzyme SusG is assisted by extracellular sugar-binding proteins (45, 46).

The *k*_cat_/*K_m_* value of *Bo*Man26B for M6 hydrolysis is close to 5000 times lower than for *Bo*Man26A (Table 2) and, for example, *Cj*Man26A (26). This implies *Bo*Man26B would be more suited to hydrolyze longer substrates, which is logical considering its outer membrane location. In contrast, *Bo*Man26A seems more suited for oligosaccharide hydrolysis, which is consistent with its periplasmic location.

*Bo*Man26A has M2 as a clear main product when hydrolyzing all tested mannan substrates. The preference for generating M2 is shared between *Bo*Man26A and its closest characterized homologues, which all are suggested to be mannobiohydrolases that are sensitive to galactose substitutions (23, 31, 47). This, together with galactomannan hydrolysis product profiles and the capability of accommodating backbone mannosyls through subsite −3 to +3, suggests that *Bo*Man26A is not a true endo-acting β-mannanase but is a β-1,4-mannobiohydrolase capable of endo-action. It preferably attacks unsubstituted or low-substituted substrates, being restricted by galactose substituents. The differences in hydrolyzing INM and LBG by the two enzymes might be explained by differences in mode of attack and progression. *Bo*Man26A may have a higher degree of processivity, but because it is restricted by galactosyl substituents, this possible processivity, if any, may be more pronounced using the unsubstituted mannan INM. This is consistent with the product profile observed for this substrate (Fig. 3*A*). So far, only one endo-β-mannanase has been suggested to be processive, *i.e.* the mannobiohydrolase studied by Tsukagoshi *et al.* (47).

*Bo*Gal36A hydrolyzes internal galactosyl decorations from galactomannans, in contrast to other GH36 α-galactosidases, including those from gut bacteria, which generally act on raffinose and similar di- and trisaccharides (34). Besides *Bo*Man26A, the predicted periplasmic transcriptional regulator (*bacova_02097*) of the *B. ovatus* β-mannan PUL is also sensitive to galactosyl substitutions and binds undecorated β-mannan oligosaccharides more efficiently (10). Thus, the probable function of *Bo*Gal36A is removal of internal galactose substitutions from galactomannan poly- or oligosaccharides produced by the less sensitive *Bo*Man26B, enabling the effective utilization of galactomannan as the carbon source.

##### Model of the Galactomannan Catabolism of B. ovatus

**FIGURE 8.**
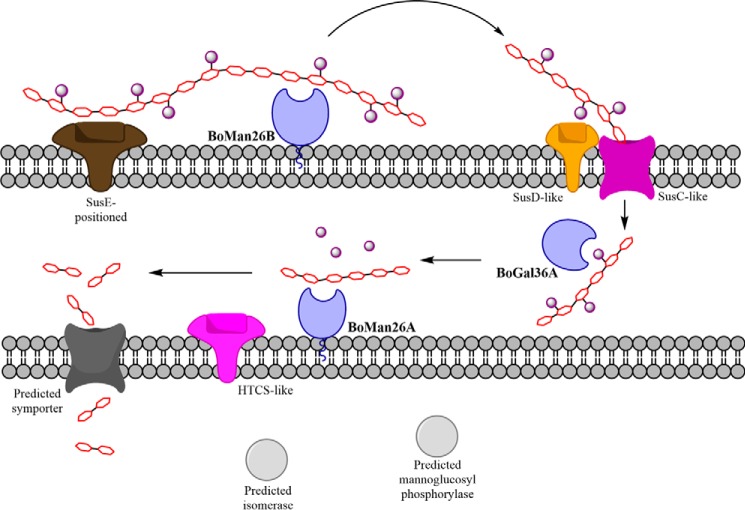
**Galactomannan degradation in *B. ovatus*.** Overview of the proposed action of the *bacova_02087–97* PUL proteins conferring galactomannan hydrolysis and utilization. The outer membrane is shown on *top*. The proteins are colored according the corresponding genes in the PUL overview in Fig. 1. The GHs studied in this paper are colored *blue* and highlighted in *bold*. SusE-positioned, SusD-like, SusC-like, and HTCS-like correspond to *bacova_02094–97*, respectively. The *gray* enzymes are those for which the genes are uncolored in Fig. 1. The predicted isomerase, symporter, and mannoglucosyl phosphorylase (corresponding to *bacova_02088–90*, respectively) have initially been assigned these functions based on a protein BLAST search, as well as Senoura *et al.* (41). The polysaccharide chain contains a mannose backbone (*red*) with galactose substitutions (*purple spheres*).

The mode of action described above is supported by the cellular location data, where *Bo*Man26B was shown to be surface-exposed, and likely associated with the outer membrane, and *Bo*Gal36A and *Bo*Man26A likely being located in the periplasm (Fig. 2). *Bo*Man26B would thus initially attack the galactomannan substrate, producing galactomanno-oligosaccharides. These would be further processed in the periplasm, first by *Bo*Gal36A to cleave off galactosyl substituents and then by *Bo*Man26A, releasing M2, as suggested by the synergy experiments. The produced M2 would possibly be internalized and further processed via a putative mannosyl-phosphorylase and an isomerase encoded by the currently studied PUL, in a similar way as proposed for the mannan catabolic pathway in *B. fragilis* (Fig. 8) (31).

##### Structure-Function Relation and Role of BoMan26A

According to the above model, *Bo*Man26A would be optimized to hydrolyze unsubstituted manno-oligosaccharides. Although the catalytic efficiency on M3 to M6 (Table 2) is considerably lower than the mannobiohydrolase *Cj*Man26C (23), it is comparable with some extracellular GH26 mannanases of environmental bacteria (27) and much higher than *Bo*Man26B. The large difference in *k*_cat_/*K_m_* values when comparing M3 with M4 hydrolysis (Table 2) suggests that *Bo*Man26A requires occupation of at least four subsites for efficient hydrolysis (−2 to +2), which is similar to *Cj*Man26C (23), whereas *Bf*Man26A requires filling of five subsites for efficient hydrolysis (31). Furthermore, the ^18^O-labeling experiments for *Bo*Man26A show that substrate mannosyls can also be accommodated in subsites −3 and +3 (Fig. 4). The ability to accommodate mannosyls in subsite −3 distinguishes *Bo*Man26A from *Cj*Man26C. In addition, *Bo*Man26A has a more open aglycone region within the +2 and any potential +3 subsite and beyond (Figs. 6 and 7).

The galactose substituent attached to the mannosyl present in the −1 subsite in the *Cj*Man26C structure fits in the *Bo*Man26A structure (Figs. 6 and 7), indicating that galactose perhaps could be accommodated in the −1 subsite. *Cj*Man26C has also been shown to not be able to accommodate galactose in the +1 subsite (23). This situation is likely also in *Bo*Man26A, based on the overlay. This could at least partly explain its sensitivity to galactose.

Based on substrate binding in *Cj*Man26C, loop 2 appears to close off the area beyond the −2 subsite in *Bo*Man26A, despite ^18^O labeling showing that *Bo*Man26A is capable of accommodating a mannosyl in the −3 subsite. The low *B*-factor of loop 2 indicates low flexibility in the crystal structure; however, a stabilizing factor may be that Asp-99 in loop 2 hydrogen bonds to His-373, the last residue in the His tag of the neighboring molecule in the crystal. Thus, loop displacement to generate a −3 subsite cannot be ruled out in solution, especially because the equivalent loop is shorter in other endo-acting β-mannanases, giving a more open active site cleft (26–28). Potential flexibility in loop 2 could be a possible explanation for the productive binding mode preference for M5 revealed by ^18^O labeling (Fig. 4); the flexibility would allow −3 occupation yet still restrict the area beyond the −2 subsite. Taken together with the more open region beyond subsite +2, this would result in higher substrate occupation of the +3 subsite but disfavor the −3 subsite. Such flexibility is also a criteria for the capability of endo-action.

The difference of backbone position between loop 8 of *Bo*Man26A and the α-helical turn in *Cj*Man26C causes a relatively small difference between the two enzymes due to the structural conservation of the side chain positions. This preserves the hydrogen bonding between loops 2 and 8 (exo-loop and α-helical turn in *Cj*Man26C), as well as the salt bridge. The higher *B*-factor and potential flexibility of loop 8, compared with the rest of the *Bo*Man26A structure, indicates that loop breathing is possible. Although the indicated flexibility in loop 8 in the *Bo*Man26A structure is similar to that seen in the α-helix turn of *Cj*Man26C, loop breathing enabling endo-activity is further indicated by the fact that equivalent positions for this loop are missing from the other two endo-acting GH26 mannanases *Cf*Man26A and *Cj*Man26A (26, 27). Taken together, the more open +3 subsite and the potential loops 2 and 8 conformational changes allowing a −3 subsite may explain the endo-activity we observed for *Bo*Man26A but was not observed for *Cj*Man26C (23).

##### Concluding Remarks

In light of the cellular location analysis and the biochemical properties of the two characterized β-mannanases, we propose a scheme of sequential action by the GHs involved in the β-mannan utilization pathway in *B. ovatus* (Fig. 8). The outer membrane-associated *Bo*Man26B initially acts on the polysaccharide galactomannan producing larger oligosaccharide fragments and mannobiose. Galactomanno-oligosaccharides are further processed in the periplasm, degalactosylated by *Bo*Gal36A and subsequently hydrolyzed into mannobiose units by the β-mannanase *Bo*Man26A.

## Experimental Procedures

### 

#### 

##### Chemicals

The following manno-oligo- and polysaccharides were purchased from Megazyme (Bray, Ireland): M2, M3, M4, M5, M6, G2M5, INM, LBG, KGM, and borohydride-reduced LBG galactomannan. Mannose was from Fluka (Steinheim, Germany). If not stated otherwise all other chemicals were from Sigma.

##### Construction of B. ovatus PUL Mutant Strains

*B. ovatus* Δ*tdk* (a strain for allelic exchange) was used as the parent strain to create mutant deletion strains. Deletions within both β-mannan-related PULs (Fig. 1) were done by allelic exchange as described previously using the 5-fluoro-2-deoxyuridine-resistant strain (Δ*tdk*) that lacks the gene for thymidine kinase (16). Primers used are listed in supplemental Table S1. A deletion of *bacova_02087–02096* (from the PUL *bacova_02087–02097*) was created, and the strain was named *B. ovatus* ΔGGM. In the same way *bacova_03400–03403* (encoding putative GH26 and SusC/D-like proteins from the PUL *bacova_03386–03406*) was deleted, generating strain *B. ovatus* Δ03400-03. A strain with both the above deletions was constructed and named *B. ovatus* ΔGGMΔ03400-03.

##### Cloning of bacova_02092 and bacova_02093 from B. ovatus

The genes *bacova_02092* and *bacova_02093* (UniProt accession numbers A7LW88 and A7LW89, respectively, and GenBank^TM^ accession numbers EDO12202.1 and EDO12203.1, respectively) were mined from the genomic sequence data of *B. ovatus* ATCC 8483. A BlastP search and multiple sequence alignment were performed. The presence of signal peptidase I and II cleavage sites was analyzed using the SignalP (48) and LipoP (49) servers, respectively, and the expressed sequences were designed to omit any signal peptides (supplemental Fig. S2).

All cloning reagents were from Thermo Scientific. The truncated *Bo*Man26A gene *bacova_02092* was amplified by PCR from the genomic DNA of *B. ovatus,* prepared as described previously (16). The PCRs (50 μl) contained MgCl_2_ (2 mm), DNA (50 ng), dNTPs (250 μm), primers (0.5 μm each, see supplemental Table S2), dimethyl sulfoxide (2%), and *Pfu* DNA polymerase (2.5 units). The amplified PCR products were double-digested by the NcoI and XhoI enzymes and cloned into the appropriate restriction sites in the pET28b+ expression vector (Novagen, Merck, Darmstadt, Germany). This generated the plasmid pB2092, where the *bacova_02092* gene was fused with a sequence coding for a C-terminal His_6_ tag. To verify the presence of the construct, the coding region of the plasmid was DNA-sequenced using T7 primers (Eurofins Genomics, Ebersberg, Germany). The plasmid was transformed into electrocompetent BL21(DE3) *Escherichia coli* cells for protein expression.

A plasmid containing the full-length *bacova_02093* was initially created (pB2093) the same way as described above using the appropriate primers (supplemental Table S2). Using pB2093 as a template, a plasmid (pB2093TR) carrying a truncated gene variant of *bacova_02093* was generated where the coding region for residues 1–19 was deleted based on LipoP (49) lipid anchor prediction (supplemental Fig. S2). Primers (supplemental Table S2) were used in PCR-mediated deletion of plasmid DNA as described previously (50) to produce the plasmid pB2093TR. The presence of the anticipated deletion product was shown by agarose gel electrophoresis and the plasmid was transformed into OneShot® TOP10 Electrocomp^TM^ *E. coli* cells (Life Technologies, Inc.), following the manufacturer's recommendations. The presence of the desired construct was verified by colony PCR using MyTaq^TM^ HS DNA polymerase (Bioline, London, UK) and T7 primers, and by DNA sequencing also using T7 primers (Eurofins Genomics, Ebersberg, Germany). The pB2093TR plasmid was transformed into chemocompetent *E. coli* BL21(DE3) cells for protein expression.

##### Glycoside Hydrolase Expression and Purification

*E. coli* BL21(DE3) cells containing the pB2092 or pB2093TR plasmid (encoding His-tagged *Bo*Man26A and *Bo*Man26B, respectively) were grown in 5 ml of Luria-Bertani medium with 30 μg/ml kanamycin at 37 °C, 150 rpm until exponential phase (OD_600_ ≈0.7). Expression was induced by adding 0.4 mm isopropyl β-d-1-thiogalactopyranoside (IPTG), and the culture was continued for an additional 2 h. The cells were dissolved in lysis buffer (50 mm NaH_2_PO_4_, 300 mm NaCl, 10 mm imidazole, pH 8) and lysed by a French pressure cell. The lysed cells were centrifuged, and the resulting supernatants were incubated with 2 ml of nickel-nitrilotriacetic acid slurry (Qiagen, Hilden, Germany) overnight with head-over-tail rotation at 4 °C before being poured into a gravity flow column with a maximum volume of 15 ml. The resulting gel bed was drained and washed three times with 4 ml of wash buffer (50 mm NaH_2_PO_4_, 300 mm NaCl, 20 mm imidazole, pH 8). The protein was eluted with elution buffer (50 mm NaH_2_PO_4_, 300 mm NaCl, 250 mm imidazole, pH 8).

The eluted fractions were evaluated by estimating their protein concentration with a Nanodrop ND-1000 spectrophotometer using absorbance at 280 nm, and the theoretical extinction coefficients (*Bo*Man26A, 89,890 m^−1^ cm^−1^, and *Bo*Man26B, 136,560 m^−1^ cm^−1^, calculated from the ProtParam ExPASy server (51)) were analyzed by SDS-PAGE. *Bo*Man26A and *Bo*Man26B were assayed for β-mannanase activity as described below. The purified fractions were pooled, and the buffer was changed to 50 mm potassium phosphate, pH 6.5, using 10-kDa molecular mass cutoff membrane filtration tubes (Vivaspin 20, Sartorius, Little Chalfont, UK) and stored at 4 °C for further analysis. The protein was mixed with 4× loading buffer (0.7 m Tris-HCl, pH 6.8, 10% glycerol, 2% SDS, 6% mercaptoethanol, and 0.05% bromphenol blue), boiled for 5 min, and run on an SDS-polyacrylamide gel (ClearPage 12% gels and running buffer, CBS Scientific, San Diego). The obtained preparations of *Bo*Man26A and *Bo*Man26B were electrophoretically homogeneous (supplemental Fig. S3). The resulting expressed proteins, including their fused His tag, had a total protein length and theoretical molecular mass of 350 residues and 40,117 Da for *Bo*Man26A and 347 residues and 39870 Da for *Bo*Man26B. *Bo*Gal36A was expressed and purified as described previously (34).

##### Cloning and Production of Sugar Binding Proteins and Affinity Gel Electrophoresis

The genes encoding Bacova_02094 (residues 23–391) and Bacova_02095 (residues 42–603) were amplified by PCR from the genomic DNA of *B. ovatus* using the primers listed in supplemental Table S2. The PCR-amplified genes were ligated into pET-28rTEV and fused to a tobacco etch virus protease (TEV)-cleavable N-terminal His_6_ tag using the NheI and XhoI restriction sites and sequenced to verify correct incorporation of the gene. pET-28rTEV is identical to pET-28a (EMD Millipore, Bedford, MA) except that the thrombin cleavage site was changed to that of TEV. The Bacova_02094-pET28rTEV and Bacova_02095-pET28rTEV plasmids were transformed into Rosetta (DE3) pLysS cells (EMD Millipore), plated onto Luria-Bertani medium with 30 μg/ml kanamycin, and grown for 16 h at 37 °C. The plates were scraped of all colonies and used to inoculate 1 liter of Terrific Broth, including 30 μg/ml kanamycin and 20 μg/ml chloramphenicol at 37 °C. Cells were grown to an OD_600_ of ∼0.6, induced with 0.5 mm IPTG, and moved to 20 °C for another 16 h. Cells were centrifuged and resuspended in 50 ml of cold His buffer (25 mm NaH_2_PO_4_, 500 mm NaCl, 20 mm imidazole, pH 7.4), adding one EDTA-free cOmplete protease inhibitor tablet (Roche Applied Science, Basel, Switzerland) and 0.1% Triton X-100. The cells were lysed on ice by sonication and centrifuged. The supernatant was loaded onto a 5-ml HiTrap Chelating HP column (GE Healthcare, Pollards Wood, UK) charged with Ni^2+^, washed with His buffer, and eluted with a 20–300 mm imidazole gradient over 70 ml. Fractions were harvested based on SDS-PAGE of the relevant elution range. TEV protease was added at a 1:50 mg ratio (TEV/protein) to remove the His tag overnight at 4 °C while dialyzing against His buffer. The cleaved protein was separated from the His-tagged TEV protease and uncleaved protein by passage back over a 5-ml HiTrap Chelating HP column. The flow-through was harvested and dialyzed against 20 mm HEPES, 100 mm NaCl, pH 7.5, before using in affinity PAGE.

Affinity PAGE was performed similarly to Cuskin *et al.* (13), with native polyacrylamide gels consisting of 10% (w/v) acrylamide in 25 mm Tris, 250 mm glycine buffer, pH 8.8. Two of the gels contained 0.5% borohydride-reduced LBG galactomannan or KGM. Eight μg of each protein were loaded, and the electrophoresis was run at 70 V for 135 min at room temperature. BT1043, a mucin *O*-glycan targeting SusD homologue, was used as a non-binding control.

##### β-Mannanase Activity Assay

The activity was measured using the standard 3,5-dinitrosalicylic acid (DNS)-reducing sugar assay as described previously (52) using 0.12 μg/ml *Bo*Man26A or 1.6 μg/ml *Bo*Man26B and 0.5% (w/v) LBG in 50 mm potassium phosphate buffer, pH 6.5. The incubation time was 15 min at 37 °C. Mannose was used to obtain a concentration standard curve. Temperature dependence was determined between 22 and 60 °C. pH dependence was carried out in the pH range 3–8 in 0.5-unit increments. 50 mm sodium citrate buffer was used for pH 3–5.5 and 50 mm sodium phosphate for pH 6–8. Temperature stability was tested for up to 24 h at 22, 30, 37, and 45 °C. The pH and temperature dependence and stability were conducted using the standard activity assay. Incubations for specific activities were done in the same way as the standard DNS assay but using the various substrates (LBG, guar gum, KGM, and INM) at 0.5% (w/v) concentration. Incubations with insoluble INM were centrifuged at 8000 × *g* for 5 min before measuring the absorbance. Specific activity units used was katal/mol, where katal was calculated as moles of produced reducing sugars/s.

##### Product Profile Analysis

The product profile of *Bo*Man26A and *Bo*Man26B was determined with the standard assay, by incubation of 18 nanokatal/ml (2.4 μg/ml for *Bo*Man26A and 18 μg/ml for *Bo*Man26B) with the following oligo- and polysaccharides: 4 mm M2–M6, 0.25% (w/v) KGM, INM, LBG, guar gum, and partially hydrolyzed guar gum (Sunfiber, Taiyo Europe, Schwelm, Germany); Avicel cellulose (Fluka, Steinheim, Germany); spruce GGM (53); and soluble birch wood β-1,4-xylan (Roth, Karlsruhe, Germany) for 15 min and 3 and 24 h in 50 mm potassium phosphate buffer, pH 6.5, at 30 °C. The mixtures were boiled for 5 min to stop the reaction and diluted with Millipore water as follows: 1:100 for oligosaccharides; 1:50 for LBG, KGM, and guar gum; and 1:10 for INM, GGM, partially hydrolyzed guar gum, Avicel, and xylan. The dilutions were analyzed using HPAEC-PAD with a CarboPac PA-200 column (Dionex, Sunnyvale, CA) to determine the product profile, as described by Morrill *et al.* (32).

##### Enzyme Kinetics

Oligosaccharide hydrolysis kinetics of *Bo*Man26A and *Bo*Man26B were measured using HPAEC-PAD and calculated using the equation from Matsui *et al.* (54), plotting ln[S_0_]/[S*_t_*] over time, where S*_t_* is the concentration at the various time points, and S_0_ is the concentration at time 0. Mixtures of 0.05 mm oligosaccharide, 0.9 nm *Bo*Man26A or 4 μm *Bo*Man26B, and 50 mm potassium phosphate buffer, pH 6.5, were incubated at 37 °C for 0, 2, 5, 7, 10, 15, and 19 min for *Bo*Man26A and 0, 5, 10, 15, 20, 25, and 30 min for *Bo*Man26B. The reaction was stopped by adding 50% NaOH to a final concentration of 0.5%. The resulting samples were run on HPAEC-PAD with a PA-200 column to determine oligosaccharide concentration. The peak areas were analyzed using a standard curve to determine the concentration decrease of the relevant oligosaccharide. The ln[S_0_]/[S*_t_*] over time was plotted, and the trend line was divided with the enzyme concentration to give *k*_cat_/*K_m_* values for each oligosaccharide (54, 55).

##### Cellular Location Analysis

For fluorescence microscopy, the parent *B. ovatus* Δ*tdk* strain and the *B. ovatus* ΔGGM mutant with an in-frame deletion of *bacova_02087–02096* (from the PUL *bacova_02087–02097*) (Fig. 1) were grown in 5 ml of *Bacteroides* minimal media, as described previously (10), containing 0.5% LBG as the sole carbon source. The cultures were grown to an OD_600_ of 0.6, then pelleted, and washed with phosphate-buffered saline (PBS). The cells were then fixed by incubation in 4.5% formaldehyde in PBS for 1.5 h, washed with PBS, and blocked for 16 h in 2% goat serum in PBS at 4 °C. Cells were then washed with PBS and stained with custom rabbit antibodies (Innovagen, Lund, Sweden) raised against purified *Bo*Gal36A, *Bo*Man26A, and *Bo*Man26B. Primary labeling of a 1:500 dilution of the rabbit antisera in 1% goat serum was performed for 2 h at room temperature, followed by centrifugation and two washes with PBS. Secondary (fluorescence) labeling was with an Alexa-Fluor® 488-conjugated goat anti-rabbit IgG secondary antibody (Molecular Probes, Thermo Fisher Scientific). Cells were mounted on agarose pads and imaged on an Olympus IX70 inverted microscope (Olympus, Tokyo, Japan).

Western blottings were conducted using the custom antibodies against *Bo*Gal36A, *Bo*Man26A, and *Bo*Man26B as primary antibodies. These blottings included lysed *B. ovatus* Δ*tdk* and ΔGGM cells and the purified enzymes. A 25-ml cell culture of each *B. ovatus* strain was grown on LBG, pelleted, and washed as above before being resuspended (1:1 by weight) in 50 mm potassium phosphate buffer, pH 6.5. A 10-μl cell suspension of appropriate dilution was added to 5 μl of 3× SDS-PAGE loading buffer (0.25 m Tris-HCl, pH 6.8, 50% glycerol, 0.3 m SDS, 0.05% bromphenol blue, 15% mercaptoethanol). The samples were boiled for 20 min and then centrifuged at 10,000 rpm for 10 min. The protein samples were prepared similarly but were boiled for 10 min. The samples were then run on an SDS-polyacrylamide gel (12% mini-PROTEAN® TGX^TM^ gels with recommended running buffer (Bio-Rad)) in duplicate, one for immunoblotting and one for Coomassie Brilliant Blue protein staining. Transfer to a Western blot membrane was performed using cold transfer run with ice for 1 h at 100 V using Immobilon®-P transfer membranes (Millipore) with a PowerPac^TM^ 300 (Bio-Rad). After transfer the membranes were blocked for 1 h at room temperature with 3% BSA in blotting buffer (20 mm Tris-HCl, pH 7.6, with 0.15 m NaCl). The membranes were then incubated with the primary antibodies generated for *Bo*Gal36A (1:2000 dilution), *Bo*Man26A (1:2000 dilution), and *Bo*Man26B (1:10000 dilution), with 3% BSA in blotting buffer. After a washing step of three times for 10 min at room temperature with blotting buffer, the membranes were incubated with goat anti-rabbit IgG conjugated with horseradish peroxidase (Agrisera, Vännäs, Sweden), as the secondary antibody at 1:10,000 dilution with 5% dry milk in blotting buffer for 1 h at room temperature. The washing step was repeated before incubating the membrane with ECL solution (1.25 mm luminol, 200 mm *p*-coumaric acid, 2.7 mm perhydrol H_2_O_2_, and 100 mm Tris-HCl, pH 8.5) and visualizing the resulting fluorescence using a multi-application gel imaging system PXi-touch (Syngene, Cambridge, UK). Optimization of the protocol was carried out by varying the NaCl concentration of the blotting buffer up to 0.5 m for each enzyme tested, and the final NaCl concentration used for each enzyme was 0.5 m for *Bo*Man26A, 0.15 m for *Bo*Man26B, and 0.3 m for *Bo*Gal36A.

##### Synergy Experiments on G2M5 and LBG with BoGal36A and BoMan26A

5 mm G2M5 was incubated with 100 nm *Bo*Gal36A and 45 nm *Bo*Man26A individually and in a mixture. 0.5% LBG was incubated with 1 μm *Bo*Gal36A and 0.45 μm *Bo*Man26A individually and in a mixture. The buffer used was 50 mm potassium phosphate buffer, pH 6.5. The amount of M2 released and galactose released in each case was quantified by HPAEC-PAD, using CarboPac PA100 and PA10 columns, respectively, after 1 h of incubation.

##### ^18^O Labeling for BoMan26A Subsite Mapping

The preferred productive binding modes of M5 for *Bo*Man26A during hydrolysis were determined according to the method described by Hekmat *et al.* (37) using MALDI-TOF MS. The enzyme was incubated with 1 mm M5 in 5 mm potassium phosphate buffer, pH 6.5, 93% [^18^O]water at 4 °C for 120 min in a total volume of 10 μl. Small aliquots of the reaction were taken at different time points (2, 15, 30, 45, and 60 min) and cocrystallized on a MALDI plate along with the matrix 2,5-dihydroxybenzoic acid. The hydrolysis products were analyzed with a 4700 Proteomics Analyzer (Applied Biosystems, Framingham, MA). The *m*/*z* peaks of saccharides were detected as K^+^ adducts. ^18^O-Labeled and -unlabeled oligosaccharides differed by 2 Da. Appropriate enzyme blanks and substrate blanks were included according to an established protocol, which also explains the determination of labeled *versus* non-labeled product ratios and the distribution of the possible binding modes (37). This involved that the product ratio was corrected for the fact that the peak for a ^16^O-saccharide carrying 2 natural ^13^C-isotopes overlaps with the peak for the same ^18^O-saccharide, lacking any ^13^C-isotope. It was also corrected for the contaminating 7% [^16^O] water.

##### Crystallization and Data Collection of BoMan26A

To optimize the protein concentration required for crystallization trials, a Pre-Crystallization Test (PCT) from Hampton Research (Aliso Viejo) was set up. The storage buffer was changed to 20 mm Tris-HCl, pH 7.5, using 10-kDa molecular mass cutoff membrane filtration tubes (Vivaspin 20, Sartorius). The protein concentrations tested were 9, 4.5, and 2.2 mg/ml. Dynamic light scattering determined the monodispersity of the sample to be above 95% before crystallization trials were begun. Based on the PCT results, vapor diffusion (sitting drop) PACT and JCSG+ screens (Molecular Dimensions, Newmarket, UK) were set up with *Bo*Man26A using a mosquito pipetting robot (TTP Labtech, Melbourne, UK) with drop sizes of 100 nl of reservoir + 100 nl of 4.5 mg/ml protein. The plates were stored at 20 °C in a Gallery 700 plate hotel (Rigaku, Sevenoaks, UK). A crystal grown under the following conditions was used for data collection: 0.1 m potassium thiocyanate, 30% (w/v) polyethylene glycol (PEG) monoethyl ether 2000 (condition G9 of the JCSG+ screen).

Data collection was carried out at 100 K with an X-ray wavelength of 1.0 Å at the I911-3 beamline of the MAX IV Laboratory (Lund, Sweden). The cryoprotectant was introduced by soaking (<5 s) and contained an additional 15% PEG400 in 50 mm MES buffer, pH 6.5. Indexing, integration of the diffraction images, scaling of the data, and generation of an MTZ file was done using the XDS suite of programs (56) and CAD from CCP4 (57). Molecular replacement was carried out using the Phenix version of Phaser-MR (58, 59) with *Cj*Man26C, PDB code 2VX4 (sequence identity 38%) (23), as the model, after which the Phenix autobuild module (60) and restrained refinements were used for further refinement, coupled with manual refinement in Coot (61). Several cycles of alternating restrained refinement, initially using Refmac5 (62) followed by Phenix (58), and Coot manual editing were carried out to finalize the models, before submitting to PDB.

## Author Contributions

H. S., N. M. K., and E. C. M. defined the overall research topic. H. S., N. M. K., E. C. M., and V. B. planned the study. C. M. B., T. R., and Y. X. performed cellular location, growth experiments, and affinity gel electrophoresis. V. B., S. K. R., J. M., H. B., and E. K. performed cloning and basic enzyme characterization. V. B., S. K. R., and E. K. performed substrate profiling and kinetics. S. K. R. and J. M. performed ^18^O labeling. S. K. R. performed synergy analysis. V. B., O. A., and D. L. performed crystallization, structure determination, and data refinement. All authors interpreted the data. V. B., S. K. R., H. S., N. M. K., and E. C. M. wrote the manuscript with input from the other authors. All authors reviewed and approved the final version of the manuscript.

## Supplementary Material

Supplemental Data

## Footnotes

4 The abbreviations used are:

GH: glycoside hydrolase
*Bo*Gal36A: GH36 α-galactosidase from *B. ovatus*
*Bo*Man26A and B: GH26 β-mannanases A and B from *B. ovatus*
*Cf*Man26A: GH26 β-mannanase A from *C. fimi*
*Cj*Man26A and C: GH26 β-mannanases A and C from *C. japonicus*
DNS: 3,5-dinitrosalicylic acid
DP: degree of polymerization
G1M4: galactosyl-mannotetraose
G2M5: digalactosyl-mannopentaose
GGM: galactoglucomannan
HPAEC-PAD: high performance anion exchange chromatography with pulsed amperometric detection
HTCS: hybrid two-component system
INM: ivory nut mannan
IPTG: isopropyl β-d-1-thiogalactopyranoside
KGM: konjac glucomannan
LBG: locust bean gum
M2: mannobiose
M3: mannotriose
M4: mannotetraose
M5: mannopentaose
M6: mannohexaose
PCT: pre-crystallization test
PEG: polyethylene glycol
PUL: polysaccharide utilization locus
r.m.s.d.: root mean square deviation
Sus: starch-utilization system
TEV: tobacco etch virus
PDB: Protein Data Bank.
