# Supplementary material for: Galactomannan Catabolism Conferred by a Polysaccharide Utilization Locus of Bacteroides ovatus: ENZYME SYNERGY AND CRYSTAL STRUCTURE OF A β-MANNANASE
Source: J Biol Chem. 2016 Nov 21;292(1):229–43. doi: 10.1074/jbc.M116.746438 (PMC5217682; doi:10.1074/jbc.M116.746438)
Supplement: Supplemental Data [file supp_292_1_229__index.html]

Galactomannan catabolism conferred by a polysaccharide utilisation locus of Bacteroides ovatus : enzyme synergy and crystal structure of a β-mannanase — Galactomannan Catabolism Conferred by a Polysaccharide Utilization Locus of Bacteroides ovatus — Galactomannan Catabolism by B. ovatus — Supplemental Data 

# Galactomannan Catabolism Conferred by a Polysaccharide Utilization Locus of *Bacteroides ovatus*

## Supplemental Data

- Supplemental data (.pdf, 3.5 MB) - Supplemental figures and tables for the manuscript.
