# Supplementary material for: Galactomannan Catabolism Conferred by a Polysaccharide Utilization Locus of Bacteroides ovatus: ENZYME SYNERGY AND CRYSTAL STRUCTURE OF A β-MANNANASE
Source: J Biol Chem. 2016 Nov 21;292(1):229–43. doi: 10.1074/jbc.M116.746438 (PMC5217682; doi:10.1074/jbc.M116.746438)
Supplement: Supplemental Data [file 10.1074_M116.746438_jbc.M116.746438-1.pdf]

Galactomannan catabolism conferred by a polysaccharide utilisation locus of *Bacteroides ovatus*: enzyme synergy and crystal structure of a GH26 β-mannanase

Viktoria Bågenholm, Sumitha K. Reddy, Hanene Bouraoui, Johan Morrill, Evelina Kulcinskaja, Constance M. Bahr, Oskar Aurelius, Theresa Rogers, Yao Xiao, Derek T. Logan, Eric C. Martens, Nicole M. Koropatkin and Henrik Stålbrand

Supplemental information

Table S1. Primers used for generation of the PUL knockout strains of *bacova_02087-02096* and *bacova_03400-03403*

PUL name	Primer name	Primer sequence (5' to 3')
bacova_02087-02096	GGM_U	GATTTCCGGCGACGGTGACG
	GGM_NU	GCGGTCGACGTCTGGCAATCGGTGGTAACC
	GGM_A	AGATGACCTTTAAGCAAACTTGAG
	GGM_B	CTCAAGTTTGCTTAAAGGTCATCTAATATTTGATAGGTTTTAT TCTGTC
	GGM_ND	GCGGGATCCCCATCGGTCATGAGAATGTAAC
	GGM_D	CGTATGAAACAGTTTGGTTATGG
bacova_03400-03403	DelGluManAlt-UpF	AGTCGTCGACAACTCCACTATGCAACCTCTTTCAGTACTTG
	DelGluManAlt-UpR	TGCATGATAAGTTTTTAAATATTAGATCGTCG
	DelGluManAlt-DnF	CGACGATCTAATATTTAAAAACTTATCATGCATCAAAGACAAC AAGTATTCTAGTATGAAAAGACTG
	DelGluManAlt-DnR	TGACTCTAGATATCAAGAAGATAAGGACTTTCCGTACACC
	DelGluManAlt-VF	GGATGGGGAGGAAAAGCTAA
	DelGluManAlt-VR	CGCTCTGTGTTATGGACTGC

Table S2. The primers used for cloning of *bacova_2092, bacova_02093,bacova_02093TR, bacova_02094* and *bacova_02095*.

Gene name	Primer name	Primer sequence (5’ to 3’)
bacova_02092	Fw *Bo*Man26A	ATACCATGGGAAGTGGAGAAACCGGTG
	Rev *Bo*Man26A	GCGCTCGAGTTTATAGAGTTCAAAATTGTCTCCGAC
bacova_02093	Fw *Bo*Man26B	GCGCCATGGCAATGAAGAATATATATAACTTTTTG
	Rev *Bo*Man26B	GCGCTCGAGTTTAAACGAAGGCAAATCAC
bacova_02093 TR	Fw *Bo*Man26BTR	GAGATATACCATGGCATGCTCTTCCTCGTCCG
	Rev *Bo*Man26BTR	CGGACGAGGAAGAGCATGCCATGGTATATCTC
bacova_02094	Fw *bacova_02094*	CTAGCTAGCCAGGACATAGTGACCTATAATG
	Rev *bacova_02094*	GGCCTCGAGTTACCGGATTTTCTTCTCAATAC
bacova_02095	Fw *bacova_02095*	GAGCGCTAGCAACGATGATGCTGCAGTCAGC
	Rev *bacova_02095*	CGCCTCGAGTTAACTCCAACCCGGATTATTG

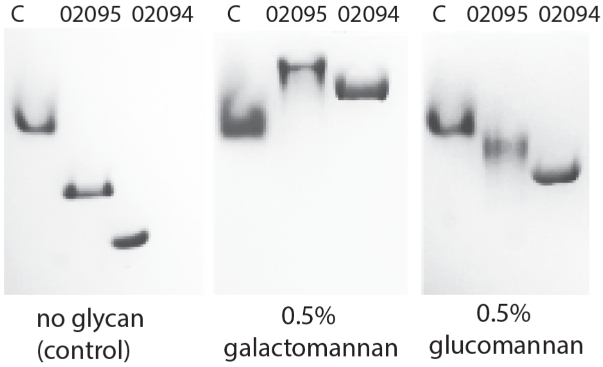

Figure S1 β-Mannan-affinity gel electrophoresis. Either 0.5% LBG galactomannan, 0.5% KGM or no glycan were included in the Native-PAGE gels. The recombinant products of gene locus *bacova_02094* (SusE positioned) or *bacova_02095* (SusD homolog) were loaded (top of the gels) and the recombinant mucus glycan binding SusD-homolog BT1043 was used as a reference protein (lane C).

A

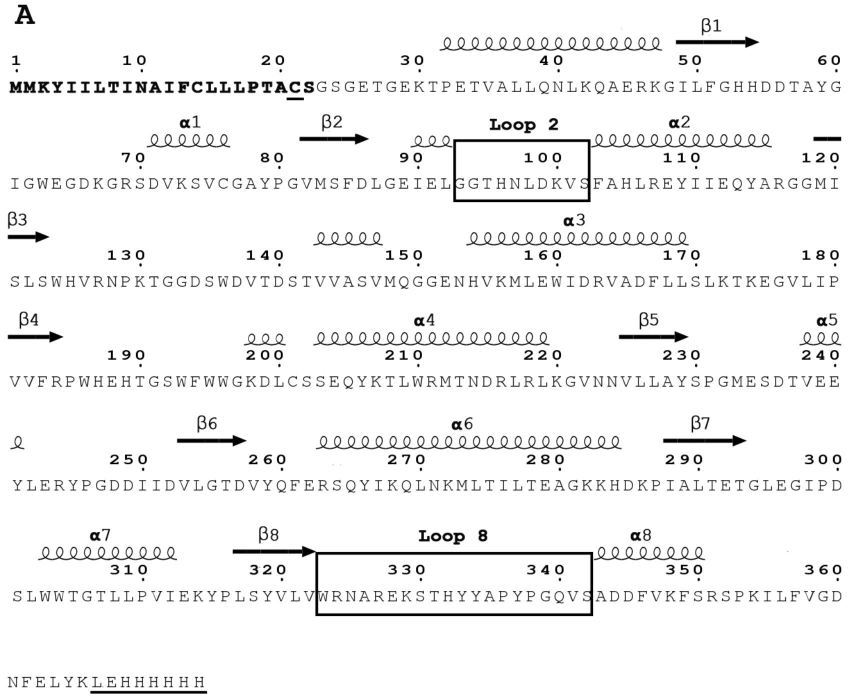

B

MKNIYNFLIYFIFCGIGAGCSSSSGEDFPAPESEPVDNSLIKKELCTEGASVEAKKVYTYLRN
CWGRKTLSSTMANVTWNVNEAIWVNRQTGKYPAIACFDYMNLPASPADWIDYNKISVVEDWWN
AGGLVAACWHWNVPVTENSSEYKCMISETDFDITKALQEGTRENEIIKADLEELAGYLLLLKQ
KNIPVIWRPLHEAAGKWFWWGKDAASYKRLWKLVYETFKQKGLNNLIWVWTSETNDRDWYPGD
AYVDIIGRDVYHKTSAAGLATDFDALKKAFPDKLIALSECGDVATIDKQLAAGAQWAWFMTWY
DYEVTKDTTAPVFNSGQHEHADKAWWNNAFGQPGVICRSDLPSFKLEHHHHHH

Figure S2 The sequences of *Bo*Man26A (A) and *Bo*Man26B (B), showing the native sequences as well as the added His-tag (underlined). The Leu codon of the His-tag replaced the native stop codon in the construct. The native sequences that were removed in the construct for each enzyme have been highlighted in bold. For *Bo*Man26A this is also the sequence predicted to be removed by signal peptidase I. The predicted lipid anchoring cyteine is underlined. Signal peptidase II is predicted to cleave immediately before this cysteine. For *Bo*Man26A the secondary structure is shown. Loops 2 and 8, as well as α-helixes and β-sheets belonging to the $\beta_8\alpha_8$-barrel structure, have been labelled.

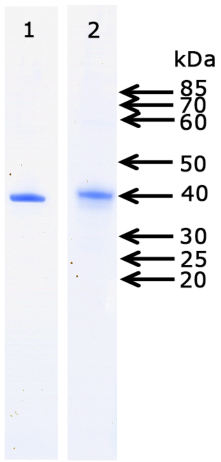

Figure S3 SDS-PAGE of purified *Bo*Man26A (1) and *Bo*Man26B (2). The sizes of the relevant bands in the ladder are labelled in kDa. The gel has been spliced to remove inappropriate concentrations. The enzymes migrated as expected at approximately 40 kDa.

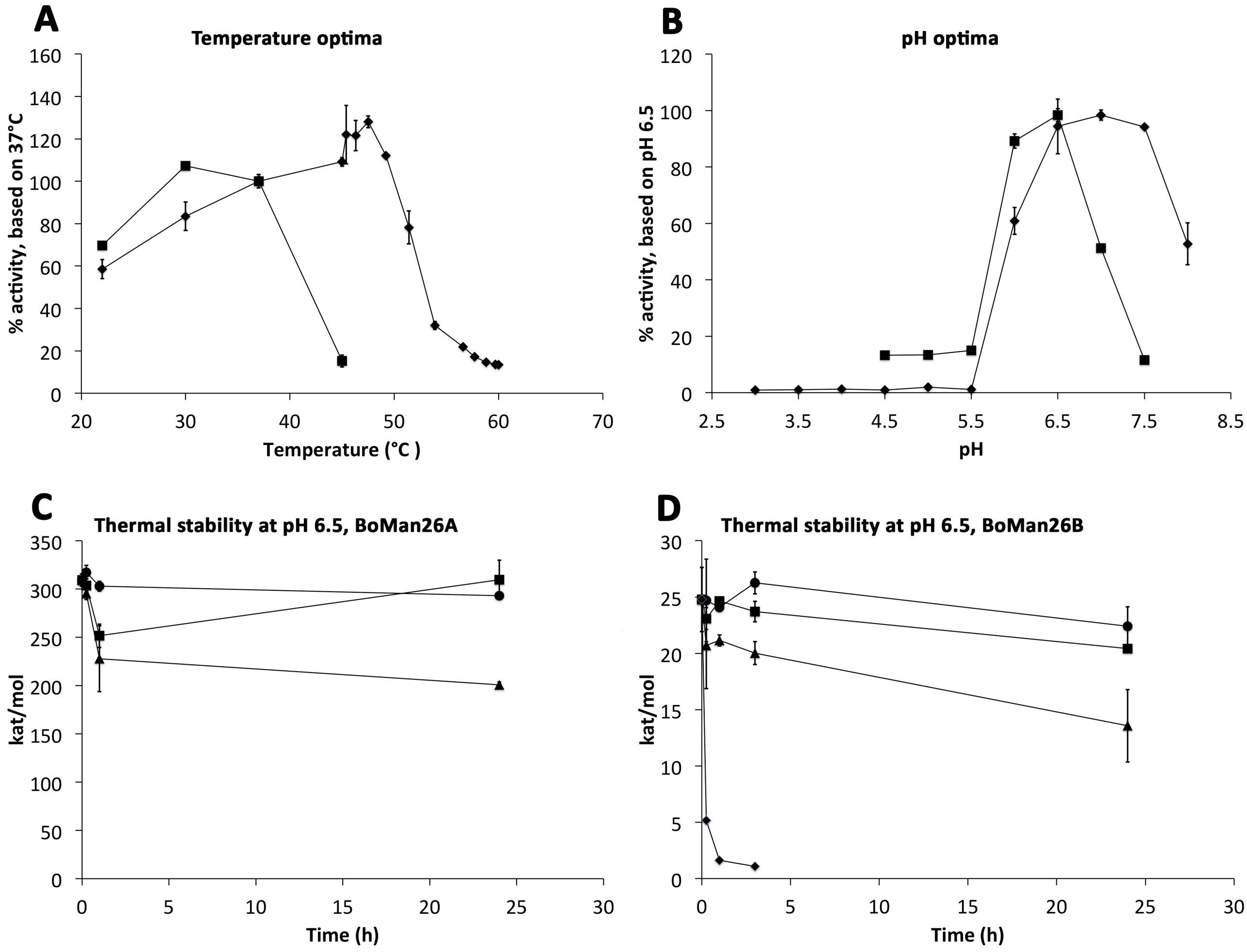

Figure S4 The effect of pH and temperature on *Bo*Man26A (◆) and *Bo*Man26B (■), determined by the DNS activity assay. **A:** Temperature optima, incubated for 15 min at temperatures between 22°C-60°C. **B:** pH optima, incubated for 15 min at 37°C between pH 3-8. **C:** *Bo*Man26A was incubated at pH 6.5 for 15 min, 1 and 24 h at 22°C (RT, ●), 37°C (■) and 45°C (▲) before running the DNS assay for 15 min at 37°C. **D:** *Bo*Man26B was incubated at pH 6.5 for 15 min, 1 h, 3h and 24 h at RT (●), 30°C (■), 37°C (▲) and 45°C (◆) before running the DNS assay for 15 min at 37°C. The error bars represent standard deviation.

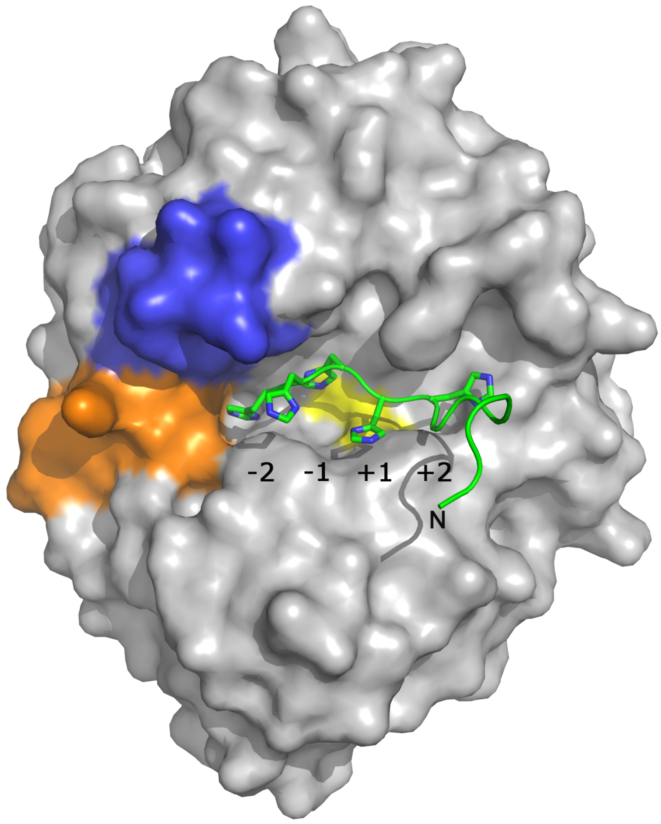

Figure S5 Surface overview of *Bo*Man26A with similar colouring to Figure 7 (loop 2 orange, loop 8 blue, catalytic residues yellow). The His-tag of the adjacent monomer in the crystal has been shown in green. The subsites and the N-terminal of the His-tag have been marked.
